# (*Z*)-4-[2-(2,4-Dimethyl­phen­yl)hydrazinyl­idene]-3-methyl­pyrazol-5(1*H*)-one

**DOI:** 10.1107/S1600536813006661

**Published:** 2013-03-13

**Authors:** B. K. Sarojini, B. J. Mohan, B. Narayana, H. S. Yathirajan, Jerry P. Jasinski, Ray J. Butcher

**Affiliations:** a Department of Chemistry, P. A. College of Engineering, Mangalore 574 153, India; b Department of Studies in Chemistry, Mangalore University, Mangalagangotri 574 199, India; c Department of Studies in Chemistry, University of Mysore, Manasagangotri, Mysore 570 006, India; d Department of Chemistry, Keene State College, 229 Main Street, Keene, NH 03435-2001, USA; e Department of Chemistry, Howard University, 525 College Street NW, Washington, DC 20059, USA

## Abstract

The mol­ecule of the title compound, C_12_H_14_N_4_O, is roughly planar, with a dihedral angle of 8.0 (8)° between the benzene and pyrazole rings, and an intra­molecular N—H⋯O hydrogen bond forms an *S*(6) ring motif. In the crystal, mol­ecules are linked into an inversion dimer by a pair of N—H⋯O hydrogen bonds, which form an *R*_2_^2^(8) ring motif.

## Related literature

For the biological activity of pyrazolo­nes, see: Amir & Kumar (2005 ▶); Rao *et al.* (2008 ▶); Samshuddin *et al.* (2011 ▶). For the radical scavenging capacity of pyrazol-5-ols, see: Sarojini *et al.* (2010 ▶). For related structures, see: Butcher *et al.* (2011 ▶); Samshuddin *et al.* (2011 ▶). For reference bond-length data, see: Allen *et al.* (1987 ▶).
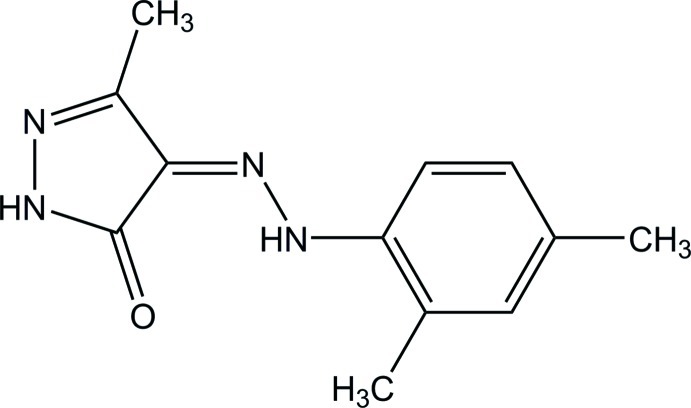


## Experimental

### 

#### Crystal data

C_12_H_14_N_4_O
*M* *_r_* = 230.27
Monoclinic, 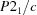

*a* = 5.2926 (2) Å
*b* = 22.1675 (6) Å
*c* = 10.0529 (3) Å
β = 101.770 (3)°
*V* = 1154.64 (6) Å^3^
*Z* = 4
Cu *K*α radiation
μ = 0.72 mm^−1^
*T* = 123 K
0.51 × 0.24 × 0.08 mm

#### Data collection

Agilent Xcalibur (Ruby, Gemini) diffractometer
Absorption correction: multi-scan (*CrysAlis PRO*; Agilent, 2012 ▶) *T*_min_ = 0.538, *T*_max_ = 0.944
4152 measured reflections
2328 independent reflections
2061 reflections with *I* > 2σ(*I*)
*R*_int_ = 0.024

#### Refinement

*R*[*F* ^2^ > 2σ(*F* ^2^)] = 0.043
*wR*(*F* ^2^) = 0.124
*S* = 1.05
2328 reflections
157 parameters
H-atom parameters constrained
Δρ_max_ = 0.34 e Å^−3^
Δρ_min_ = −0.27 e Å^−3^

### 

Data collection: *CrysAlis PRO* (Agilent, 2012 ▶); cell refinement: *CrysAlis PRO* ; data reduction: *CrysAlis RED* (Agilent, 2012 ▶); program(s) used to solve structure: *SHELXS97* (Sheldrick, 2008 ▶); program(s) used to refine structure: *SHELXL97* (Sheldrick, 2008 ▶); molecular graphics: *SHELXTL* (Sheldrick, 2008 ▶); software used to prepare material for publication: *SHELXTL*.

## Supplementary Material

Crystal structure: contains datablock(s) global, I. DOI: 10.1107/S1600536813006661/is5254sup1.cif

Structure factors: contains datablock(s) I. DOI: 10.1107/S1600536813006661/is5254Isup2.hkl

Supplementary material file. DOI: 10.1107/S1600536813006661/is5254Isup3.cml

Additional supplementary materials:  crystallographic information; 3D view; checkCIF report

## Figures and Tables

**Table 1:** Hydrogen-bond geometry (Å, °)

*D*—H⋯*A*	*D*—H	H⋯*A*	*D*⋯*A*	*D*—H⋯*A*
N2—H2*A*⋯O1^i^	0.88	1.95	2.8233 (15)	172
N4—H4*D*⋯O1	0.88	2.00	2.7286 (15)	139

Symmetry code: (i) 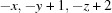.

**Fig. 1.**
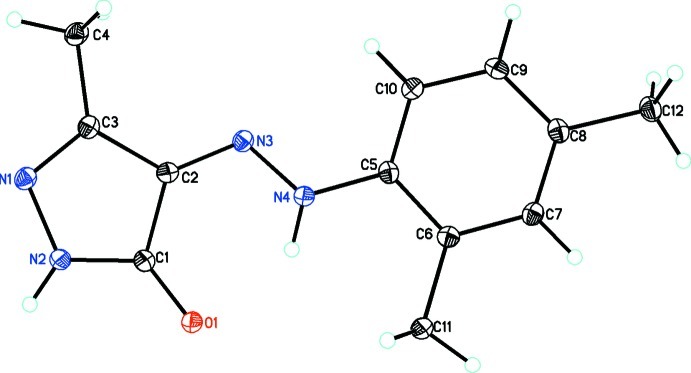
Molecular structure of the title compound showing the atom labeling scheme and 30% probability displacement ellipsoids.

**Fig. 2.**
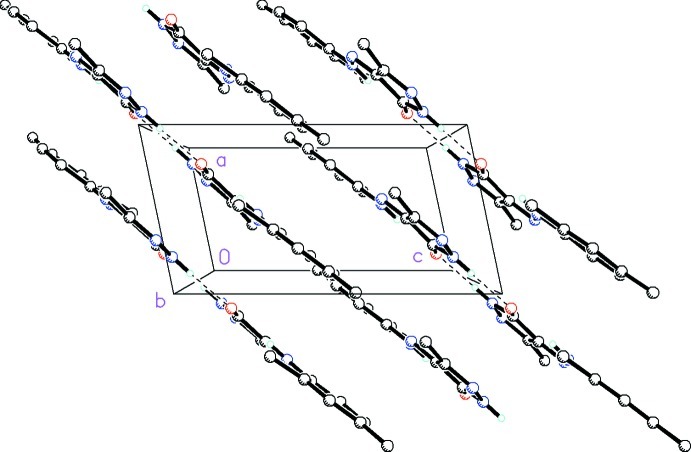
Packing diagram of the title compound viewed along the b axis. Dashed lines indicate intermolecular N—H···O hydrogen bonds which form R22(8) ring motifs linking the molecule into dimers. H atoms not involved in hydrogen bonding have been removed for clarity.

## supplementary crystallographic information

### Comment

In view of high medicinal value such as anti-inflammatory, analgesic (Amir & Kumar, 2005), antimicrobial (Samshuddin *et al.*, 2011) and antiproliferative activity (Rao *et al.* 2008) of pyrazolones, it was thought worthwhile to synthesize compounds based on pyrazolone derivatives. In addition, the radical scavenging capacity and molecular binding of various derivatives of pyrazol-5-ols were reported (Sarojini *et al.*, 2010). Also, the crystal structures of some of the related pyrazoles *viz.* 3,5-bis(4-bromophenyl)-1-phenyl-4,5-dihydro-1*H*-pyrazole (Samshuddin *et al.*, 2011) and 5-bis(4-methylphenyl)-1-phenyl-4,5-dihydro-1*H*-pyrazole (Butcher *et al.*, 2011) have been reported. In view of the importance of pyrazolones, we report herein the crystal structure of the title compound, C_12_H_14_N_4_O, (I).

The asymmetric unit of (I) consists of a 3-methyl-1*H*-pyrazol-5(4*H*)-one group bonded to 2,4-(dimethylphenyl)hydrazone in a nearly planar conformation (Fig. 1). The mean plane of the benzene ring is twisted by 8.0 (8)° from that of the pyrazole ring. Bond lengths are in normal ranges (Allen *et al.*, 1987). In the crystal, intermolecular N2—H2A···O1 hydrogen bonds (Table 1) which form *R*^2^_2_(8) ring motifs linking the molecule into dimers are observed (Fig. 2). An intramolecular N4—H4D···O1 hydrogen bond is also present.

### Experimental

Ethyl 2-[2-(2,4-dimethylphenyl)hydrazinylidene]-3-oxobutanoate (2.62 g, 0.01 mol) and hydrazine hydrate (0.75 ml, 0.015 mol) were refluxed in acetic acid for 4–6 h. Then the reaction mixture was cooled to room temperature and the solid product was filtered off. Single crystals were grown by slow evaporation from a mixture of petroleum ether and ethyl acetate (1:3 v/v) (m.p. 421–423 K).

### Refinement

All of the H atoms were placed in their calculated positions and then refined using the riding model with Atom—H lengths of 0.95 Å (CH), 0.98 Å (CH_3_) or 0.88 Å (NH). Isotropic displacement parameters for these atoms were set to 1.2 (CH, NH) or 1.5 (CH_3_) times *U*_eq_ of the parent atom.

### Crystal data

**Table:**

C_12_H_14_N_4_O	*F*(000) = 488
*M**_r_* = 230.27	*D*_x_ = 1.325 Mg m^−^^3^
Monoclinic, *P*2_1_/*c*	Cu *K*α radiation, λ = 1.54178 Å
Hall symbol: -P 2ybc	Cell parameters from 2088 reflections
*a* = 5.2926 (2) Å	θ = 4.0–75.3°
*b* = 22.1675 (6) Å	µ = 0.72 mm^−^^1^
*c* = 10.0529 (3) Å	*T* = 123 K
β = 101.770 (3)°	Long plate, colourless
*V* = 1154.64 (6) Å^3^	0.51 × 0.24 × 0.08 mm
*Z* = 4	

### Data collection

**Table:**

Agilent Xcalibur (Ruby, Gemini) diffractometer	2328 independent reflections
Radiation source: Enhance (Cu) X-ray Source	2061 reflections with *I* > 2σ(*I*)
Graphite monochromator	*R*_int_ = 0.024
Detector resolution: 10.5081 pixels mm^-1^	θ_max_ = 75.5°, θ_min_ = 4.0°
ω scans	*h* = −5→6
Absorption correction: multi-scan (*CrysAlis PRO*; Agilent, 2012)	*k* = −27→26
*T*_min_ = 0.538, *T*_max_ = 0.944	*l* = −11→12
4152 measured reflections	

### Refinement

**Table:**

Refinement on *F*^2^	Primary atom site location: structure-invariant direct methods
Least-squares matrix: full	Secondary atom site location: difference Fourier map
*R*[*F*^2^ > 2σ(*F*^2^)] = 0.043	Hydrogen site location: inferred from neighbouring sites
*wR*(*F*^2^) = 0.124	H-atom parameters constrained
*S* = 1.05	*w* = 1/[σ^2^(*F*_o_^2^) + (0.0699*P*)^2^ + 0.3531*P*] where *P* = (*F*_o_^2^ + 2*F*_c_^2^)/3
2328 reflections	(Δ/σ)_max_ < 0.001
157 parameters	Δρ_max_ = 0.34 e Å^−^^3^
0 restraints	Δρ_min_ = −0.27 e Å^−^^3^

### Special details

**Table:**

Geometry. All esds (except the esd in the dihedral angle between two l.s. planes) are estimated using the full covariance matrix. The cell esds are taken into account individually in the estimation of esds in distances, angles and torsion angles; correlations between esds in cell parameters are only used when they are defined by crystal symmetry. An approximate (isotropic) treatment of cell esds is used for estimating esds involving l.s. planes.
Refinement. Refinement of *F*^2^ against ALL reflections. The weighted *R*-factor *wR* and goodness of fit *S* are based on *F*^2^, conventional *R*-factors *R* are based on *F*, with *F* set to zero for negative *F*^2^. The threshold expression of *F*^2^ > σ(*F*^2^) is used only for calculating *R*-factors(gt) *etc*. and is not relevant to the choice of reflections for refinement. *R*-factors based on *F*^2^ are statistically about twice as large as those based on *F*, and *R*-factors based on ALL data will be even larger.

### Fractional atomic coordinates and isotropic or equivalent isotropic displacement parameters (Å^2^)

**Table:**

	*x*	*y*	*z*	*U*_iso_*/*U*_eq_	
O1	0.18460 (18)	0.45530 (5)	0.89208 (10)	0.0213 (2)	
N1	0.2909 (2)	0.61180 (5)	0.90296 (12)	0.0212 (3)	
N2	0.1729 (2)	0.55870 (5)	0.93665 (12)	0.0206 (3)	
H2A	0.0590	0.5581	0.9893	0.025*	
N3	0.5733 (2)	0.50000 (5)	0.73044 (12)	0.0190 (3)	
N4	0.5362 (2)	0.44146 (5)	0.73009 (12)	0.0196 (3)	
H4D	0.4265	0.4266	0.7766	0.024*	
C1	0.2517 (3)	0.50879 (6)	0.88022 (14)	0.0183 (3)	
C2	0.4380 (3)	0.53140 (6)	0.80200 (14)	0.0186 (3)	
C3	0.4473 (3)	0.59579 (6)	0.82398 (14)	0.0196 (3)	
C4	0.6109 (3)	0.63998 (6)	0.76933 (16)	0.0242 (3)	
H4A	0.6007	0.6793	0.8126	0.036*	
H4B	0.5497	0.6438	0.6709	0.036*	
H4C	0.7903	0.6260	0.7885	0.036*	
C5	0.6657 (3)	0.40171 (6)	0.65796 (14)	0.0187 (3)	
C6	0.6331 (3)	0.33981 (6)	0.67717 (14)	0.0190 (3)	
C7	0.7561 (3)	0.29989 (6)	0.60384 (14)	0.0202 (3)	
H7A	0.7338	0.2578	0.6145	0.024*	
C8	0.9106 (3)	0.31954 (7)	0.51552 (14)	0.0208 (3)	
C9	0.9423 (3)	0.38169 (7)	0.50101 (15)	0.0226 (3)	
H9A	1.0493	0.3960	0.4425	0.027*	
C10	0.8203 (3)	0.42275 (6)	0.57057 (14)	0.0216 (3)	
H10A	0.8416	0.4648	0.5590	0.026*	
C11	0.4740 (3)	0.31736 (6)	0.77591 (15)	0.0229 (3)	
H11A	0.2952	0.3310	0.7465	0.034*	
H11B	0.5452	0.3333	0.8667	0.034*	
H11C	0.4783	0.2732	0.7785	0.034*	
C12	1.0424 (3)	0.27468 (7)	0.43960 (16)	0.0259 (3)	
H12A	0.9883	0.2337	0.4578	0.039*	
H12B	1.2299	0.2783	0.4698	0.039*	
H12C	0.9946	0.2829	0.3419	0.039*	

### Atomic displacement parameters (Å^2^)

**Table:**

	*U*^11^	*U*^22^	*U*^33^	*U*^12^	*U*^13^	*U*^23^
O1	0.0210 (5)	0.0191 (5)	0.0251 (5)	−0.0010 (4)	0.0078 (4)	−0.0011 (4)
N1	0.0195 (6)	0.0181 (6)	0.0257 (6)	0.0016 (4)	0.0038 (5)	0.0009 (5)
N2	0.0192 (6)	0.0197 (6)	0.0246 (6)	0.0005 (4)	0.0081 (5)	−0.0002 (4)
N3	0.0184 (5)	0.0176 (6)	0.0204 (6)	0.0011 (4)	0.0024 (4)	0.0006 (4)
N4	0.0190 (5)	0.0182 (6)	0.0226 (6)	−0.0010 (4)	0.0068 (4)	−0.0005 (4)
C1	0.0155 (6)	0.0201 (7)	0.0188 (6)	0.0016 (5)	0.0021 (5)	0.0004 (5)
C2	0.0168 (6)	0.0190 (6)	0.0193 (6)	0.0003 (5)	0.0023 (5)	0.0022 (5)
C3	0.0176 (6)	0.0185 (6)	0.0214 (6)	0.0034 (5)	0.0012 (5)	0.0014 (5)
C4	0.0228 (7)	0.0182 (6)	0.0320 (8)	0.0026 (5)	0.0066 (6)	0.0036 (6)
C5	0.0163 (6)	0.0203 (7)	0.0190 (6)	0.0006 (5)	0.0023 (5)	−0.0024 (5)
C6	0.0161 (6)	0.0209 (7)	0.0190 (6)	−0.0023 (5)	0.0015 (5)	−0.0010 (5)
C7	0.0184 (6)	0.0183 (7)	0.0224 (7)	−0.0018 (5)	0.0005 (5)	−0.0023 (5)
C8	0.0156 (6)	0.0242 (7)	0.0213 (6)	0.0004 (5)	0.0010 (5)	−0.0043 (5)
C9	0.0199 (6)	0.0269 (7)	0.0223 (7)	−0.0018 (6)	0.0073 (5)	−0.0003 (6)
C10	0.0228 (7)	0.0187 (6)	0.0233 (7)	−0.0010 (5)	0.0049 (5)	0.0002 (5)
C11	0.0257 (7)	0.0190 (6)	0.0255 (7)	−0.0037 (5)	0.0085 (6)	−0.0016 (5)
C12	0.0223 (7)	0.0256 (7)	0.0309 (7)	−0.0011 (6)	0.0079 (6)	−0.0080 (6)

### Geometric parameters (Å, º)

**Table:**

O1—C1	1.2504 (17)	C5—C6	1.4013 (19)
N1—C3	1.3075 (19)	C6—C7	1.395 (2)
N1—N2	1.4059 (16)	C6—C11	1.5112 (18)
N2—C1	1.3475 (18)	C7—C8	1.394 (2)
N2—H2A	0.8800	C7—H7A	0.9500
N3—N4	1.3125 (16)	C8—C9	1.399 (2)
N3—C2	1.3135 (18)	C8—C12	1.5084 (19)
N4—C5	1.4052 (18)	C9—C10	1.385 (2)
N4—H4D	0.8800	C9—H9A	0.9500
C1—C2	1.4692 (18)	C10—H10A	0.9500
C2—C3	1.4437 (19)	C11—H11A	0.9800
C3—C4	1.4850 (19)	C11—H11B	0.9800
C4—H4A	0.9800	C11—H11C	0.9800
C4—H4B	0.9800	C12—H12A	0.9800
C4—H4C	0.9800	C12—H12B	0.9800
C5—C10	1.397 (2)	C12—H12C	0.9800
			
C3—N1—N2	106.63 (11)	C7—C6—C11	121.39 (13)
C1—N2—N1	113.29 (12)	C5—C6—C11	120.92 (12)
C1—N2—H2A	123.4	C8—C7—C6	122.40 (13)
N1—N2—H2A	123.4	C8—C7—H7A	118.8
N4—N3—C2	115.29 (12)	C6—C7—H7A	118.8
N3—N4—C5	122.19 (12)	C7—C8—C9	118.22 (13)
N3—N4—H4D	118.9	C7—C8—C12	120.53 (13)
C5—N4—H4D	118.9	C9—C8—C12	121.24 (13)
O1—C1—N2	128.34 (13)	C10—C9—C8	121.07 (13)
O1—C1—C2	127.52 (12)	C10—C9—H9A	119.5
N2—C1—C2	104.13 (12)	C8—C9—H9A	119.5
N3—C2—C3	127.04 (13)	C9—C10—C5	119.43 (13)
N3—C2—C1	127.81 (12)	C9—C10—H10A	120.3
C3—C2—C1	105.13 (12)	C5—C10—H10A	120.3
N1—C3—C2	110.81 (12)	C6—C11—H11A	109.5
N1—C3—C4	122.32 (13)	C6—C11—H11B	109.5
C2—C3—C4	126.86 (13)	H11A—C11—H11B	109.5
C3—C4—H4A	109.5	C6—C11—H11C	109.5
C3—C4—H4B	109.5	H11A—C11—H11C	109.5
H4A—C4—H4B	109.5	H11B—C11—H11C	109.5
C3—C4—H4C	109.5	C8—C12—H12A	109.5
H4A—C4—H4C	109.5	C8—C12—H12B	109.5
H4B—C4—H4C	109.5	H12A—C12—H12B	109.5
C10—C5—C6	121.18 (13)	C8—C12—H12C	109.5
C10—C5—N4	121.68 (13)	H12A—C12—H12C	109.5
C6—C5—N4	117.14 (12)	H12B—C12—H12C	109.5
C7—C6—C5	117.68 (13)		
			
C3—N1—N2—C1	−0.01 (15)	N3—N4—C5—C10	−7.1 (2)
C2—N3—N4—C5	179.75 (12)	N3—N4—C5—C6	172.99 (12)
N1—N2—C1—O1	179.22 (13)	C10—C5—C6—C7	−1.3 (2)
N1—N2—C1—C2	−0.17 (15)	N4—C5—C6—C7	178.63 (11)
N4—N3—C2—C3	177.63 (12)	C10—C5—C6—C11	177.61 (12)
N4—N3—C2—C1	−0.2 (2)	N4—C5—C6—C11	−2.45 (19)
O1—C1—C2—N3	−0.9 (2)	C5—C6—C7—C8	1.0 (2)
N2—C1—C2—N3	178.50 (13)	C11—C6—C7—C8	−177.90 (12)
O1—C1—C2—C3	−179.13 (13)	C6—C7—C8—C9	0.2 (2)
N2—C1—C2—C3	0.28 (14)	C6—C7—C8—C12	179.27 (12)
N2—N1—C3—C2	0.20 (15)	C7—C8—C9—C10	−1.1 (2)
N2—N1—C3—C4	−178.85 (12)	C12—C8—C9—C10	179.80 (13)
N3—C2—C3—N1	−178.55 (13)	C8—C9—C10—C5	0.8 (2)
C1—C2—C3—N1	−0.30 (15)	C6—C5—C10—C9	0.4 (2)
N3—C2—C3—C4	0.5 (2)	N4—C5—C10—C9	−179.51 (12)
C1—C2—C3—C4	178.70 (13)		

### Hydrogen-bond geometry (Å, º)

**Table:**

*D*—H···*A*	*D*—H	H···*A*	*D*···*A*	*D*—H···*A*
N2—H2*A*···O1^i^	0.88	1.95	2.8233 (15)	172
N4—H4*D*···O1	0.88	2.00	2.7286 (15)	139

Symmetry code: (i) −*x*, −*y*+1, −*z*+2.
